# Sleep Quality and Related Clinical Manifestations in Huntington Disease

**DOI:** 10.3390/jpm12060864

**Published:** 2022-05-25

**Authors:** Sabrina Maffi, Eugenia Scaricamazza, Simone Migliore, Melissa Casella, Consuelo Ceccarelli, Ferdinando Squitieri

**Affiliations:** 1 Huntington and Rare Diseases Unit, Fondazione IRCCS Casa Sollievo della Sofferenza Hospital, 71013 San Giovanni Rotondo, Italy; sabrina.maffi@gmail.com (S.M.); eugenia138@hotmail.com (E.S.); sim.migliore@gmail.com (S.M.); 2 Italian League for Research on Huntington (LIRH) Foundation, 00185 Rome, Italy; melissa.casella@lirh.it (M.C.); consuelo.ceccarelli@lirh.it (C.C.)

**Keywords:** Huntington disease, sleep, PSQI, ISI, questionnaires, ENROLL-HD, UHDRS, PBA, depression, cognitive functions

## Abstract

(1) Background: Sleep patterns are frequently disrupted in neurodegenerative disorders such as Huntington disease (HD); however, they are still poorly understood, especially their association with clinic features. Our study aimed to explore potential correlations between sleep features and motor, cognitive, behavioural and functional changes in manifest HD subjects. (2) Methods: We enrolled 42 patients who were assessed by the Pittsburgh Sleep Quality Index (PSQI) and Insomnia Severity Index (ISI) questionnaires; clinical features were evaluated by the validated ENROLL-HD platform assay, including the Unified Huntington’s Disease Rating Scale (UHDRS) and the Problem Behaviours Assessment Short Form (PBA-s). (3) Results: We found a significant association between the patients’ perception of sleep abnormalities and scores of impaired independence, cognitive and motor performances. Specifically, sleep efficiency (PSQI—C4 subscores) and the use of sleep medications (PSQI—C6 subscores) seem to be more frequently associated with the severity of the disease progression. (4) Conclusion: sleep abnormalities represent an important part of the HD clinical profile and can impair patients’ quality of life by affecting their level of independence, cognition performance and mental well-being.

## 1. Introduction

Huntington disease (HD) is a rare, genetic, neurodegenerative disorder affecting people worldwide, with a prevalence of approximately 10 cases per 100,000 people and higher rates in North America, Western Europe, the Middle East and Australia [1,2]. It is caused by an expanded CAG repeat mutation, which contributes to neurological aging at onset [3]. The neurotoxic effects induced by the mutant protein progressively spread through the nervous system causing a dysfunctional process that primarily affects brain connections and striatum [4] and then extends to additional brain structures, including the cortex [5].

There is growing evidence regarding how other relevant non-motor symptoms contribute to disease progression; among them, a major role is played by sleep and circadian abnormalities (i.e., poor sleep quality and greater severity of insomnia) that may start early in the patient’s life [6,7,8]. Polysomnography studies highlighted dysregulation in multiple aspects of sleep in HD patients even though no specific patterns have been identified to date [9,10,11]. The most frequent alterations concern insomnia (i.e., the delay in falling asleep and the repeated night-time awakenings), reduced sleep efficiency, REM-sleep alteration and increased motor activity during sleepiness [11,12]. Moreover, patients’ sleep disturbance is related to clinical profile severity, neuropsychiatric symptoms and cognitive functions decline as well as caudate nucleus atrophy [13]. Furthermore, an increase in sleep spindle density has also been reported in HD [10,14].

In general, sleep is a complex, adaptive and physiological process that is central to cognition and behavioural regulation in healthy subjects as well as in patients affected by neurodegenerative diseases. As a consequence, impaired sleep quality and quantity may affect executive functions [15,16], learning and memory consolidation [17,18] and emotional processing [19,20] even in healthy subjects. Sleep quality and quantity may also contribute to the appearance and/or worsening of psychiatric symptoms such as anxiety, depression, irritability and apathy [21,22]. In addition, sleep disorders are frequently associated with neurodegenerative diseases such as Alzheimer’s disease (AD), progressive supranuclear palsy (PSP) and corticobasal degeneration [23,24,25]. An association between sleep disorders, abnormal executive functions, and a deficit of memory, attention, language, and visuospatial abilities has also been described in Parkinson’s disease (PD) [26,27]. For instance, there is growing evidence that sleep abnormalities may accelerate the progression of the neurodegenerative process and may contribute to the disease pathogenesis of AD and other related dementias [28].

By collecting data from self-reported questionnaires, our study aimed to highlight the potential relationship between the perception of HD subjects’ sleep disturbance and the clinical variables collected in the context of ENROLL-HD, the largest international observational study on HD [29].

## 2. Materials and Methods

### 2.1. Patients’ Population

Our sample consisted of 42 consecutive participants (20F) with genetically confirmed HD diagnoses, a Unified HD Rating Scale (UHDRS) Total Motor Score (TMS) >5 and a Diagnostic Confidence Level (DCL) ≥3. All participants were included in the observational ENROLL-HD platform by LIRH Foundation site, and data were retrospectively analysed. General inclusion criteria were: normal or corrected to normal visual acuity, normal colour vision and appropriate cognitive capacity. Patients with a significant cognitive impairment (MMSE < 20) were excluded in order to guarantee a reliable participation in the self-reported questionnaires. Patients with greater motor impairment were eventually helped in ticking the questionnaire response boxes by their caregivers. Sample details are included in Table 1.

### 2.2. Clinical Measures

All participants completed two self-reported questionnaires during the onsite visit: the Insomnia Severity Index (ISI) and the Pittsburgh Sleep Quality Index (PSQI), which investigated the presence of insomnia in the last month and the overall quality of sleep, respectively. The ISI is a short self-reported questionnaire that investigates the severity of insomnia, its interference with daytime functioning, the level of discomfort caused by sleep alteration and the degree of satisfaction with one’s own sleep patterns. The total score ranges from a minimum of 0 to a maximum of 28. Subscales were also calculated: Severity (ISI1), Sleep Subjective Satisfaction (ISI2) and Impact on Daily Activities (ISI3). For each index, high scores indicate poor sleep performance/satisfaction, or greater impact on daily activities.

The PSQI is a short self-reported questionnaire for the evaluation of sleep quality referring to the last four weeks. The PSQI provides a total score and 7 composite indexes: subjective sleep quality, C1; sleep latency, C2; sleep duration, C3; habitual sleep efficiency, C4; sleep disturbances, C5; use of sleep medications, C6; daytime dysfunction, C7. Higher scores indicate greater impairment of the relative components.

With the aim of evaluating the clinical profile, all subjects underwent a UHDRS assessment that included: TMS, Total Functional Capacity (TFC), Independence Scale (IS) and Functional Assessment (FA). Behavioural features were assessed with a Problem Behavioural Assessment Short Form (PBA-s), Hamilton Anxiety and Depression Scale (HADS-SIS) self-reported questionnaire, and a comprehensive cognitive evaluation consisting of Symbol Digit Modality Test (SDMT), Verbal Fluency (Semantic SVF and Phonemic FVF), Stroop Colour Naming (SCN), Stroop Word Reading (SWR), Stroop Interference Test (SIT), and Trail Making Test (TMT-A and TMT-B) in accordance with ENROLL-HD guidelines [30]. All assessments were performed on the same day.

### 2.3. Ethical Approval

This study conforms with the World Medical Association Declaration of Helsinki. It received approval by the local Institutional Review Board (prot. number 102/14, approved on 28 May 2014). All participants signed an informed consent before filling out the questionnaires.

### 2.4. Statistical Analysis

We performed a Pearson’s correlation analysis; the alpha level was fixed to ≤0.05. All statistical analyses were performed using IBM SPSS Statistics for Macintosh, version 25.0 (IBM Corp., Armonk, NY, USA). We entered the sleep subscales into the statistical model (ISI1, ISI2, ISI Tot, PSQI C1, C2, C3, C4, C5, C6, C7, PSQI Global) as well as motor (TMS), functional (TFC, IS, FA), behavioural (PBA subscores, HADS-SIS subscores), and cognitive variables (SDMT, SVF, FVF, SCN, SWR, SIT, TMT-A, TMT-B).

## 3. Results

### 3.1. Sleep and Clinical Scores of Independence and Motor Impairment

We found a significant association between clinical scores of impaired independence and motor performances and perception of sleep abnormalities: lower TFC scores were associated with a greater severity of insomnia (ISI1 *r* = −0.354, *p* = 0.025; ISI2 *r* = −0.393, *p* = 0.012; ISI3, *r* = −0.348, *p* = 0.028, ISI total *r* = −0.401, *p* = 0.010) and greater impairment of sleep quality (C4 *r* = −0.423, *p* = 0.005, C6 *r* = −0.324, *p* = 0.037); lower FA scores were associated with a greater severity of insomnia (ISI1 *r* = −0.381, *p* = 0.015, ISI2 *r* = −0.404, *p* = 0.010, ISI3 *r* = −0.366, *p* = 0.020, ISI total *r* = −0.420, *p* = 0.007) and greater impairment of sleep quality (C4 *r* = −0.460, *p* = 0.002, C6 *r* = −0.441, *p* = 0.003, PSQI global *r* = −0.336, *p* = 0.030); lower IS scores were associated with a greater severity of insomnia (ISI3 *r* = −0.371, *p* = 0.018, ISI2 *r* = −0.400, *p* = 0.011, ISI total *r* = −0.393, *p* = 0.012) and greater impairment of sleep quality (C4 *r* = −0.403, *p* = 0.008, C6 *r* = −0.376, *p* = 0.014); higher TMS scores were associated with a greater impairment of sleep quality (C4 *r* = 0.383, *p* = 0.012) (Table 2). The associations with quality of sleep concerned the subscores C4 and C6, which evaluated the habitual sleep efficiency and the use of sleep medications, respectively.

### 3.2. Sleep and Cognitive Scores

We found the following significant associations: lower SDMT scores were associated with a greater impairment of sleep quality (C4 *r* = −0.350, *p* = 0.025); lower SVF scores were associated with a greater impairment of sleep quality (C4 *r* = −0.449, *p* = 0.003, C6 *r* = −0.309, *p* = 0.046); lower FVF scores were associated with a greater severity of insomnia (ISI1 *r* = −0.359, *p* = 0.037) and greater impairment of sleep quality (C4 *r* = −0.460, *p* = 0.005); lower SCN scores were associated with a greater impairment of sleep quality (C4 *r* = −0.407, *p* = 0.008); lower SWR scores were associated with a greater impairment of sleep quality (C4 *r* = −0.433, *p* = 0.004); lower SIT scores were associated with a greater impairment of sleep quality (C6 *r* = −0.383, *p* = 0.017); lower TMT-B scores were associated with a greater impairment of sleep quality (C3 *r* = −0.414, *p* = 0.014; C4 *r* = −0.501, *p* = 0.002) (Table 3). The abnormal quality of sleep was specifically associated with C3, C4 and C6 subscores, which concern sleep duration, habitual sleep efficiency and use of sleep medications, respectively.

### 3.3. Sleep and Behavioural Scores

We found the following significant associations: greater scores of self-reported depression were associated with a greater severity of insomnia and impairment of sleep quality (HAD-SIS-D with ISI3, *r* = 0.625, *p* < 0.001, HAD-SIS-D with ISI2 *r* = 0.467, *p* = 0.014, HAD-SIS-D with ISI total *r* = 0.551, *p* = 0.003; HAD-SIS-D with C1 *r* = 0.449, *p* = 0.016; HAD-SIS-D with C5 *r* = 0.488, *p* = 0.008, HAD-SIS-D with C6 *r* = 0.469, *p* = 0.012; HAD-SIS-D with C7 *r* = 0.675, *p* < 0.001; HAD-SIS-D with PSQI total *r* = 0.602, *p* < 0.001); greater rater-reported depression was associated with a greater impairment of sleep quality (PBA-D with C5, *r* = 0.395, *p* = 0.010; PBA-D with C6, *r* = 0.318, *p* = 0.040); greater rater-reported apathy was associated with a greater impairment of sleep quality (PBA-A and C4 *r* = 0.312, *p* = 0.044; PBA-A and C6 *r* = 0.373, *p* = 0.015); greater self-reported irritability was associated with a greater impairment of sleep quality (HAD-SIS-I with C5 r 0.415, *p* = 0.028); greater self-reported anxiety was associated with a greater impairment of sleep quality (HAD-SIS and C7 *r* = 0.414, *p* = 0.032) (Table 4). The association of psychiatric features with the quality of sleep concerned the subscores C1, C4, C5, C6 and C7, which evaluate the subjective perception of sleep quality, habitual sleep efficiency, sleep disturbances, use of sleep medications and daytime dysfunction, respectively.

**Figure 1:**
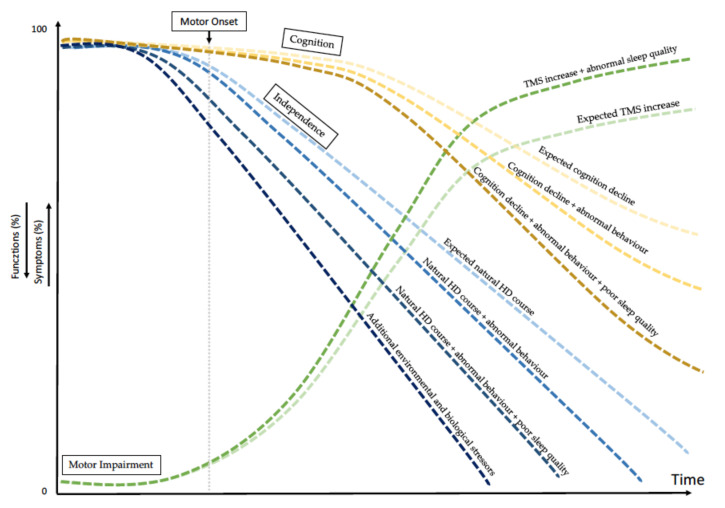
Variables affecting natural history of Huntington disease. The graph shows the simulated timing of HD progression due to the abnormal quality of patients’ sleep together with additional stressors, potentially affecting the natural HD history (i.e., biological and environmental factors). Among biological factors are intra- and extra-*HTT* gene modifiers. Among environmental factors are education, cognitive reserve and quality of sleep. All together, these several factors affect motor and cognitive declines separately or in combination and affect patients’ behaviour. TMS = Total Motor Score.

We summarised all findings concerning the potential effects of the altered quality of sleep and behaviour on cognition, movement and independence in a graph (Figure 1).

## 4. Discussion

Sleep abnormalities and circadian disorders contribute to the spectrum of clinical manifestations in several neurodegenerative diseases, including HD, AD and PD [31]. They impair cognition, behaviour and independence, thus severely affecting the quality of life of both patients and caregivers. 

Our study highlighted some significant associations between clinical manifestations and qualitative sleep parameters in HD. In our analysis, mild insomnia and abnormal general sleep efficiency were inversely correlated with patients’ loss of independence, scored by UHDRS-FA, UHDRS-IS and TFC, the latter is a Food and Drug Administration (FDA)approved measure of functional capacity. Our data suggest that the greater the sleep disturbance, the more severe the disease manifestations. Specifically, sleep efficiency (PSQI—C4 subscore) and the use of sleep medications (PSQI—C6 subscore) seem to be more frequently associated with the severity of disease progression (Figure 1).

Interestingly, our findings also highlighted a correlation between impaired sleep quality and worse cognitive performances, particularly with regard to executive functions. Our data show that chronic sleep inefficiency (PSQI—C4 subscore) negatively correlated with almost all cognitive scores; in other words, the lower the sleeping time percentage in relation to the time spent in bed, the worse the efficiency in information processing speed (SDMT), alternating attention (TMT-B) and verbal fluency tasks (SVF, FVF). Our data are in line with a pattern of alterations that is typical of frontal–subcortical network damage and with other findings that highlight the association between sleep deprivation and poorer performance on executive function tasks [32]. In addition, we also observed that cognitive performance may be negatively influenced by the use of sleep medications (PSQI—C6 subscore), further recommending the cautious use of neuroleptics to treat HD. Our findings underline the importance of a concomitant, non-pharmacological management of behavioural disturbances, including sleep difficulties, in HD and other neurodegenerative diseases [33,34,35]. For instance, a targeted cognitive behavioural psychotherapy may represent an additional resource to manage the first stages of HD. Therefore, studies investigating the efficacy of these interventions are highly recommended [36].

Another interesting finding of our study is the strong association between depression, both self-reported by the patients and reported by the raters, and several sleep scores, including sleep disturbance (PSQI—C5 subscore). Our analysis shows that the score of self-reported depression also correlates with insomnia (ISI scores), poor quality of sleep (PSQI—C1 subscore) and impact on daily life activities (PSQI—C7 subscore). In addition, other findings highlight an association between the apathy reported by the rater and the anxiety and irritability reported by the patients with a decreased quality of sleep. Altogether, these data highlight the occurrence of a possible bidirectional relationship between sleep and behaviour disturbance in HD that certainly requires further investigation. These findings are in line with previous reports on other clinical conditions concerning the negative impact of sleep abnormalities on psychiatric symptoms where a bidirectional relationship between impaired sleep and depression were also documented [37,38,39]. Our data are also in line with the evidence that sleep deprivation may solicit irritability, depression, memory and learning impairment [40], slower reaction times, and metabolic alterations and hormonal imbalances [41,42] as it was also described in healthy individuals.

Our observations have important implications, especially given the documented tendency of suicide in HD. In a recent review [43], the authors investigated the link between sleep disorders (e.g., nightmares and insomnia complaints) and suicidal behaviour in depressed patients and underscored how sleep disorders may trigger and/or aggravate suicidal behaviour. Considering the high risk of suicide occurring in HD from the premanifest stage of life [44,45,46], further analyses are needed to eventually disclose possible associations between HD sleep abnormalities and suicidal ideation.

Future analyses should also make use of objective measurements of the circadian rhythm (e.g., an actigraph) in order to further evaluate advanced stages, which have motor and cognitive impediments with self-reporting instruments.

We recently described an association with specific patterns of changes in behaviour and cognition in manifest HD [47]. Our current findings add another piece to the puzzle by underlying a possible association of apathy, depression and irritability with abnormal sleep. It also demonstrates that specific patterns of behavioural and cognitive-associated changes may affect the development of HD and raises the interesting question of when such associations may start in a patient’s life. For instance, specific analyses on the quality of sleep in premanifest cohorts may address new insights into HD natural history. Finally, circadian rhythms and sleep–wake cycles are known to be disrupted in HD, thus encouraging the investigation of these processes, which interfere with biological changes, such as hormonal release, cardiovascular function, body temperature and feeding behaviour.

In our study, we observed an association between sleep disturbances and common HD features. There is growing evidence that sleep abnormalities may accelerate the progression of neurodegenerative diseases and may even play a role in their pathogeneses [28]. Similarly, we believe that altered sleep quality may influence the progression of HD by affecting cognition. Psychiatric manifestations, in general, are unpredictable in HD because they might be potentially affected by environmental factors such as the quality of sleep (Figure 1) [47,48].

Our study has several limitations and strengths. One limitation concerns the limited size of our cohort. However, despite this limitation, we selected a homogeneous early-stage HD cohort, which underwent regular clinical analyses by validated and standardised assays within the context of a global study, such as ENROLL-HD. Another limitation concerns the variable degree of patients’ awareness of their own symptoms [13], which may have potentially affected the self-reported methodology of some responses in the questionnaires [49]. However, we did our best to ensure the caregiver’s assistance to a patient, if and when required. One strength of our preliminary study is the significant association of the altered sleep quality with TFC, an FDA-validated measure of functional capacity, currently used in the Phase 3 *pridopidine* trial [43].

## 5. Conclusions

In conclusion, sleep quality seems to affect a number of clinical manifestations and, ultimately, the severity of HD course. Our data highlight a correlation between sleep alterations and cognitive performance, behavioural abnormalities and loss of independence, thus corroborating the idea that sleep quality may represent another environmental factor affecting disease development. Dysregulated sleep may influence a wide spectrum of heterogeneous clinical manifestations of HD, which require careful counselling and therapeutic management. Further investigations, including studies on biological bases of sleep disruption in HD as well as new technological assessments by objective digital measures, are strongly needed and expected to improve the interpretation of the relationship between sleep disruption and the progression of disease severity.

## Figures and Tables

**Table 1:** Patients’ scores across the outcome variables.

	Manifest HD Cohort	
Subjects (number)	Male	22 (52.4%)
	Female	20 (47.6%)
Age (years)	Median	44.59 (±10.06)
	Range	28–64
Education (years)	Median	13.11 (±4.19)
	Range	6–22
Expanded CAG repeat	Median (SD)	44.16 (±2.98)
	Range	40–52
Disease burden score *	Median	451.9 (±103.96)
	Range	234.58–718.96
TMS	Median	30.23 (±15.64)
	Range	5–59
TFC	Median	10.14 (±2.6)
	Range	6–13
MMSE	Median	26.9 (±2.62)
	Range	20–30

Total Motor Score (TMS), Total Function Capacity (TFC), Mini Mental State Examination (MMSE). * Disease burden score = age × (CAG length—33.66).

**Table 2:** Pearson’s correlations (and related level of significance) between motor/functional variables and sleep subscales.

Sleep Subscales	TFC		FA		TMS		IS	
	*r*	*p*	*r*	*p*	*r*	*p*	*r*	*p*
ISI1	−0.354 *	0.025	−0.381 *	0.015	0.306	0.055	−0.293	0.067
ISI2	−0.393 *	0.012	−0.404 **	0.010	0.257	0.109	−0.371 *	0.018
ISI3	−0.348 *	0.028	−0.366 *	0.020	0.232	0.150	−0.400 **	0.011
ISI Tot	−0.401 *	0.010	−0.420 **	0.007	0.286	0.073	−0.393 *	0.012
PSQI C4	−0.423 **	0.005	−0.460 **	0.002	0.383 *	0.012	−0.403 **	0.008
PSQI C6	−0.324 *	0.037	−0.441 **	0.003	0.214	0.173	−0.376 *	0.014
PSQI Global	−0.291	0.061	−0.336 *	0.030	0.233	0.137	−0.284	0.069

Insomnia Severity Index—Severity (ISI1), Insomnia Severity Index—Sleep Subjective Satisfaction (ISI2), Insomnia Severity Index—Impact on Daily Activities (ISI3), Insomnia Severity Index Total (ISI Tot), Pittsburgh Sleep Quality Index—Habitual Sleep Efficiency (C4), Pittsburgh Sleep Quality Index—Use of Sleep Medications (C6), Total Functional Capacity (TFC), Functional Assessment (FA), Independence Scale (IS). * *p* < 0.05, ** *p* < 0.01.

**Table 3:** Pearson’s correlations (and related level of significance) between cognitive performance and sleep subscales.

	SDMT		SVF		FVF		SCN		SWR		SIT		TMT-B	
	*r*	*p*	*r*	*p*	*r*	*p*	*r*	*p*	*r*	*p*	*r*	*p*	*r*	*p*
ISI1	−0.084	0.611	−0.184	0.255	−0.359 *	0.037	−0.213	0.187	−0.126	0.465	−0.126	0.465	−0.039	0.827
ISI2	−0.199	0.225	−0.180	0.267	−0.248	0.158	−0.200	0.215	−0.221	0.195	−0.335 *	0.046	−0.124	0.493
ISI3	−0.199	0.225	−0.285	0.074	−0.279	0.110	−0.302	0.058	−0.335 *	0.046	−0.221	0.195	−0.106	0.557
ISI Tot	−0.180	0.272	−0.238	0.139	−0.319	0.066	−0.261	0.103	−0.254	0.135	−0.254	0.135	−0.101	0.576
PSQI C1	0.083	0.606	0.052	0.742	−0.201	0.239	0.090	0.570	0.092	0.583	0.092	0.583	−0.171	0.326
PSQI C2	−0.119	0.459	−0.204	0.194	−0.211	0.217	−0.165	0.296	−0.125	0.454	−0.125	0.454	−0.017	0.925
PSQI C3	−0.039	0.809	−0.133	0.400	−0.254	0.135	−0.149	0.345	−0.019	0.911	−0.019	0.911	−0.414 *	0.014
PSQI C4	−0.350 *	0.025	−0.449 **	0.003	−0.460 **	0.005	−0.407 **	0.008	−0.274	0.095	−0.274	0.095	−0.501 **	0.002
PSQI C5	0.046	0.774	−0.140	0.378	−0.162	0.345	−0.104	0.513	0.058	0.728	0.058	0.728	0.000	1.000
PSQI C6	−0.268	0.090	−0.309 *	0.046	−0.126	0.465	−0.213	0.176	−0.386 *	0.017	−0.386 *	0.017	−0.221	0.202
PSQI C7	0.068	0.672	0.003	0.987	−0.044	0.798	0.051	0.751	0.116	0.489	0.116	0.489	0.211	0.223
PSQI GLOBAL	−0.152	0.343	−0.272	0.081	−0.317	0.060	−0.211	0.181	−0.153	0.359	−0.153	0.359	−0.257	0.136

Insomnia Severity Index—Severity (ISI1), Insomnia Severity Index—Sleep Subjective Satisfaction (ISI2), Insomnia Severity Index—Impact on Daily Activities (ISI3), Insomnia Severity Index Total (ISI Tot), Pittsburgh Sleep Quality Index—Subjective Sleep Quality (C1); Pittsburgh Sleep Quality Index—Sleep Latency (C2); Pittsburgh Sleep Quality Index—Sleep Duration (C3); Pittsburgh Sleep Quality Index—Habitual Sleep Efficiency (C4); Pittsburgh Sleep Quality Index—Sleep Disturbances (C5); Pittsburgh Sleep Quality Index—Use of Sleep Medications (C6); Pittsburgh Sleep Quality Index—Daytime Dysfunction (C7), Symbol Digit Modality Test (SDMT), Semantic Verbal Fluency (SVF), Phonemic Verbal Fluency (FVF), Stroop Colour Naming (SCN), Stroop Word Reading (SWR) and Stroop Interference Test (SIT), Trail Making Test -B (TMT-B). * *p* < 0.05, ** *p* < 0.01.

**Table 4:** Pearson’s correlations (and related level of significance) between mood and behavioural aspects and sleep subscale.

	Apathy (PBA)		Depression (HADS)		Irritability (HADS)	
	*r*	*p*	*r*	*p*	*r*	*p*
ISI2	0.233	0.148	0.467 *	0.014	−0.012	0.954
ISI3	0.281	0.079	0.625 ***	<0.001	0.009	0.964
ISI Tot	0.272	0.089	0.551 **	0.003	−0.003	0.990
PSQI C1	0.064	0.686	0.449 *	0.016	0.130	0.510
PSQI C4	0.312 *	0.044	0.339	0.077	−0.016	0.934
PSQI C5	0.140	0.375	0.488 **	0.008	0.415 *	0.028
PSQI C6	0.373 *	0.015	0.469 *	0.012	0.198	0.312
PSQI C7	0.057	0.718	0.675 ***	<0.001	0.335	0.082
PSQI Global	0.266	0.089	0.602 ***	<0.001	0.280	0.148

Insomnia Severity Index—Sleep Subjective Satisfaction (ISI2), Insomnia Severity Index—Impact on Daily Activities (ISI3), Insomnia Severity Index Total (ISI Tot), Pittsburgh Sleep Quality Index—Subjective Sleep Quality (C1), Pittsburgh Sleep Quality Index—Habitual Sleep Efficiency (C4); Pittsburgh Sleep Quality Index—Sleep Disturbances (C5); Pittsburgh Sleep Quality Index—Use of Sleep Medications (C6); Pittsburgh Sleep Quality Index—Daytime Dysfunction (C7), Problem Behavioural Assessment (PBA), Hamilton Anxiety and Depression Scale (HADS). * *p* < 0.05, ** *p* < 0.01, *** *p* < 0.001.

## Data Availability

The data that support the findings of this study are available from the corresponding author upon reasonable request.
